# The Millennium Development Goals Fail Poor Children: The Case for Equity-Adjusted Measures

**DOI:** 10.1371/journal.pmed.1000062

**Published:** 2009-04-28

**Authors:** Daniel D. Reidpath, Chantal M. Morel, Jeffrey W. Mecaskey, Pascale Allotey

**Affiliations:** 1 Centre for Public Health Research, Brunel University, Uxbridge, United Kingdom; 2 LSE Health, London School of Economics, London, United Kingdom; 3 Save the Children UK, London, United Kingdom

## Abstract

Daniel Reidpath and colleagues use the fourth Millennium Development Goal (MDG) as an illustrative example to highlight the potential to neglect equity in the race to achieve the MDGs.

**Summary Points**

- The Millennium Declaration is a statement of principles about the kind of future that world governments seek; a future that they envisage to be more equitable and more responsive to the socially most vulnerable.
- The Millennium Development Goals represent the operational targets by which we may judge their actions.
- The reduction of the U5MR by two-thirds by 2015 is one of the Millennium Development Goals (MDG4).
- The reduction in U5MR can, however, be achieved through a diversity of policy interventions, some of which could leave the children of the poor worse off. A celebrated MDG4 success can, thus, be a Millennium Declaration failure.
- Health policy informed by composite outcome measures that take account of both the U5MR and the distribution of the burden of mortality across social groups would help to overcome this.

## Introduction

The spirit of the Millennium Declaration is to address the health and development needs of society's most vulnerable and least served [1]. Issues of equity form a key principle:

We recognize that, in addition to our separate responsibilities to our individual societies, we have a collective responsibility to uphold the principles of human dignity, equality and equity at the global level. As leaders we have a duty therefore to all the world's people, especially the most vulnerable and, in particular, the children of the world, to whom the future belongs [1].

The Declaration is operationalised through eight Millennium Development Goals (MDGs), each of which provides the blueprint and targets for addressing prioritised social needs. A tension arises, however, between the broad social principles of the Declaration, and the refined targets of the MDGs. Using the fourth MDG as an illustrative example, we highlight the potential to neglect equity in the race to achieve the set targets. MDG4 aims to reduce the under-five child mortality rate (U5MR) by two-thirds by the year 2015 [2]. Arguments analogous to those presented here are, however, applicable to all the MDGs.

As a goal, in and of itself, reducing child mortality by two-thirds is laudable. There is a significant strain, however, between an isolated MDG4-oriented outcome, and outcomes that also take account of the broader spirit of the Declaration. The problem arises because MDG4 is presented in terms of the raw, average U5MR for a country. While this makes for simple reporting, the figure masks distributional information about which parts of society contribute most (or least) to the magnitude of that rate. In other words, the measure is equity-blind, unable to distinguish between a fair and an unfair social distribution of the burden of under-five mortality [3],[4]. As a consequence, countries can achieve MDG4 (an apparent success), but fail to address the problem of under-five child mortality amongst their society's most vulnerable groups (a Millennium Declaration failure). Even where this tension has been officially recognised, there has been a failure to alter the indicators of MDG4 success [5].

To illustrate some of the issues, we present data on a hypothetical country with a U5MR around 200—that is, 200 child deaths per 1,000 live births per year. This child mortality rate, while very high, is not unheard of, and was recorded in Chad in 2004 [6], Malawi in 2000 [7], and Burkina Faso in 2003 [8]. From empirical data we know that child mortality is not equally distributed across the population and that the wealthiest groups tend to experience the lowest child mortality rates. Thus, the average U5MR may be 200, but the rate amongst the wealthiest will often be a half or a quarter of that amongst the poorest [9].

## Exploring Equity, Equality, and U5MR

If a country has unequal U5MRs across all the wealth groups, there is a situation of health inequality. Inequality, however, does not necessarily mean inequity (i.e., unfairness). Inequality refers simply to variation in the distribution of the health outcome within groups in the population (or between populations) [3],[10]. If a health inequality arises because of socially modifiable factors, then the issue is not simply variation in the distribution of health outcomes (inequality), but one of an underlying unfairness (inequity). An observable variation in the U5MR across wealth groups in a society suggests a health inequity. Similarly, inequities may arise from social differences other than wealth, such as gender, ethnicity, or religion [5]. Again, for the purposes of illustration we focus on wealth, but the analysis could potentially be applied to any of these social factors as well as to the inter-relationships between them.

For our hypothetical country to achieve its MDG4 target it must, by 2015, reduce its child mortality rate from 200 deaths to 66.7 deaths per 1,000 live births. For argument's sake let us assume that regardless of wealth, all groups experience an identical UM5R (i.e., equality). Table 1 shows the U5MR for this hypothetical country today and three possible U5MR outcomes by 2015, assuming it achieves its MDG4 target. The rate is shown for each quintile of wealth in the population as well as the average rate for the population as a whole. As a point of contrast the distribution of the U5MR in Peru (1996) is shown in the last row of the table [11].

**Table 1:** Three policy options for a hypothetical country with a U5MR of 200 today to achieve a two-thirds reduction in child mortality by 2015.

Potential Policy Objectives	Hypothetical Breakdown of U5MR by Wealth Quintiles					
	Poorest	Q2	Q3	Q4	Wealthiest	Average
Today	200	200	200	200	200	200
2015: QR1	66.7	66.7	66.7	66.7	66.7	66.7
2015: QR5	100	90	69	55	20	66.7
2015: QR10	200	55	34	25	20	66.7
Peru (QR5)	110	76.2	48	44.1	22.1	68.4

The U5MR varies across the quintiles of wealth, with each policy option showing a different quintile ratio (QR1, QR5, and QR10). Peru, with a quintile ratio of 5 in 1996, is shown as a point of contrast [11].

A multitude of policy options exist for achieving an MDG4 “success”, and each option relies on underlying social choices that will affect how different wealth groups bear the relative burden of child mortality. We consider three such options. The first policy option is to reduce the U5MR equally for each quintile of wealth in the hypothetical country. This maintains the equality of the burden of mortality across the wealth quintiles. One commonly used measure of equality is the ratio of the mortality rate in the poorest quintile over the wealthiest quintile—i.e., the quintile ratio (QR). The closer the ratio is to unity (QR1), the nearer the country is to equality of child mortality outcomes between the richest and poorest in society. A second policy option is to ignore the poorest quintile entirely and focus with decreasing effort on the wealthiest down to the second poorest quintile. This strategy can also reduce overall child mortality to the MDG4 target of 66.7, but it creates an enormous inequality between the outcomes for the wealthiest and the poorest in the society, with the poorest dying at a rate 10 times greater than the wealthiest (QR10). Because the inequality is a manifestation of the social patterning of mortality related to wealth, this also represents a substantial health inequity. There is no country with a quintile ratio as extreme as QR10, and something in the middle is more plausible (QR5). Even this third option, QR5, carries a substantial burden for the poorest in society, but nonetheless achieves MDG4. Indeed, the outcome of the third policy option is remarkably close to Peru's 1996 child mortality rate of 68.4, with a QR of 4.98 [11]. The outcome of each policy options is shown in Table 1.

Notwithstanding the fact that QR1, QR5, and QR10 all achieve MDG4 and are thus MDG “success” stories, we would argue that on a number of levels QR5 and QR10 represent significant policy failures. They achieve the stated development goal, by leaving the most vulnerable sectors of society to bear the greatest burden of child mortality, clearly violating the spirit of the Millennium Declaration. Specifically they ignore the second paragraph of the Declaration (quoted above) and corrupt the intention to “spare no effort to free our fellow men, women and children from the abject and dehumanizing conditions of extreme poverty” [1].

**Figure 1:**
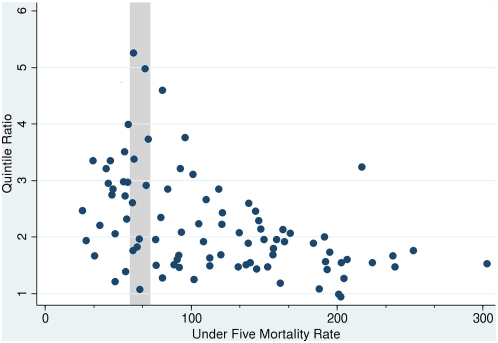
Recent U5MRs and quintile ratios for 56 countries. Data source: http://go.worldbank.org/T6LCN5A340.

Figure 1 shows a scatter plot of child mortality rates against the quintile ratio data reported by the World Bank for 56 low- and middle-income countries. These data were extracted by the authors from World Bank Country Reports. Each point represents an actual country at a particular point in recent time; some countries (for which there are more than a single year's data) appear more than once. There is considerable diversity in both the mortality rates and the quintile ratios. The column marked by the vertical grey band isolates countries with a child mortality rate around 67.7; i.e., these are countries that have already achieved the 2015 target sought by our hypothetical country described earlier. The inequity as measured by the quintile ratio in U5MR outcome, however, varies between around 1 (equity and equality) and 5 (substantial inequity and substantial inequality). Although the overall mortality rate for these countries is approximately the same, it is difficult to argue that as policy outcomes they are equally successful.

Achieving greater equity in under-five mortality is largely a matter of targeting investment and spreading resources to boost supply and demand for services particularly amongst the poor and socially excluded. While some countries may already place a high level of importance on health equity and mobilise resources accordingly, many do not. Given that the poorest populations tend also to be the hardest to reach, and hold marginal political weight, there is often little incentive for governments to prioritise their needs.

## An Equity-Adjusted Measure

Even when it is agreed that equity is an important dimension against which to evaluate a health outcome [5], two questions become inevitable. The first question relates to the trade-off between (i) lowering the mortality rate and (ii) minimising the inequity. We have seen in the U5MR data that improving one dimension (the U5MR outcome) need not improve the other (the equity outcome); and conversely, improving equity need not improve mortality (see for instance [12]). If both mortality and equity are regarded as important policy goals, then to formalise the trade-off, an objective function needs to be developed that reduces the two-dimensional problem to a single dimension, which can be maximised [13]. “There is”, as one health economist noted, “no escaping an implicit conversion [of a multidimensional evaluation] to a scalar value because it is impossible to maximize more than a single dimension at a time” [13]. The question is, thus, what form should that objective function take?

Assuming that such a function can be derived, for countries with identical child mortality rates it would identify QR1 as a healthier population than QR5, which in turn would be identified as a healthier population than QR10. It would, in effect, be an equity-adjusted measure of child mortality, analogous to adjusting life expectancy by disability in deriving a single measure of health-adjusted life expectancy.

The second question this raises is one of cost. There is a presumed efficiency/equity trade-off. Even if equity is judged to be an important dimension against which to evaluate health outcomes, what cost is one prepared to bear to achieve it?

## Conclusion

There are convincing arguments (amongst which we count the above) that in the health policy arena in particular, a composite indicator combining information about the distribution of U5MR as well as the rate itself would contribute significantly to reorienting the global health agenda. In the absence of such measures, policy failures can be readily counted as successes. The focus on the average or raw rate in MDG4, without regard to the social distribution of the burden of under-five mortality, will likely result in resource allocation being driven by expedience and lead to an increasing inequity. Evidence suggests that this is the current situation [5],[14].

Midway through the target reporting period, it is critical to take stock of and understand the exact nature of the progress being reported towards 2015. Equity is acknowledged as a significant issue [5], but without appropriate indicators of achievement, it can only ever be a talking point. An equity-adjusted measure of under-five child mortality would not suit all purposes, but it could encourage better resource distribution and, more generally, balance the desirable properties of child mortality reduction against the desirable properties of health equity. It would also better capture the spirit of the Millennium Declaration than would the equity-blind, average child mortality rate described in MDG4. Given this analysis, it is also important to examine the extent to which the other MDGs suffer from being “equity blind”.

## Abbreviations

MDG: Millennium Development Goal
QR: quintile ratio
U5MR: under-five mortality rate

## References

[1] United Nations (2000). United Nations Millennium Declaration.. http://www.un.org/millennium/declaration/ares552e.htm.

[2] United Nations (2008). The UN Millennium Development Goals.. http://www.un.org/millenniumgoals/.

[3] Reidpath DD, Allotey P (2007). Measuring global health inequity.. Int J Equity Health.

[4] Reidpath DD (2005). Population health. More than the sum of the parts?. J Epidemiol Community Health.

[5] UN Millennium Project (2005). Who's got the power? Transforming health systems for women and children. Task Force on Child Health and Maternal Health.. http://www.unmillenniumproject.org/documents/TF4Childandmaternalhealth.pdf.

[6] Gwatkin DR, Rutstein S, Johnson K, Suliman E, Wagstaff A (2007). Socio-economic differences in health, nutrition, and population: Chad. World Bank.. http://siteresources.worldbank.org/INTPAH/Resources/400378-1178119743396/chad.pdf.

[7] Gwatkin DR, Rutstein S, Johnson K, Suliman E, Wagstaff A (2007). Socio-economic differences in health, nutrition, and population: Malawi. World Bank.. http://siteresources.worldbank.org/INTPAH/Resources/400378-1178119743396/malawi.pdf.

[8] Gwatkin DR, Rutstein S, Johnson K, Suliman E, Wagstaff A (2007). Socio-economic differences in health, nutrition, and population: Burkina Faso. World Bank.. http://siteresources.worldbank.org/INTPAH/Resources/400378-1178119743396/burkinafaso.pdf.

[9] Braveman P, Gruskin S (2003). Defining equity in health.. J Epidemiol Community Health.

[10] Gwatkin DR, Rutstein S, Johnson K, Suliman E, Wagstaff A (2007). Socio-economic differences in health, nutrition, and population within developing countries: An overview. World Bank.. http://siteresources.worldbank.org/INTPAH/Resources/IndicatorsOverview.pdf.

[11] Gwatkin DR, Rutstein S, Johnson K, Suliman E, Wagstaff A (2007). Socio-economic differences in health, nutrition, and population: Peru. World Bank.. http://siteresources.worldbank.org/INTPAH/Resources/400378-1178119743396/peru.pdf.

[12] Razzaque A, Streatfield PK, Gwatkin DR (2007). Does health intervention improve socioeconomic inequalities of neonatal, infant and child mortality? Evidence from Matlab, Bangladesh.. Int J Equity Health.

[13] Phillips CV (2000). An economic analysis of human subjects research ethics; Characterizing the subjects rights – social benefits tradeoff. Boston University School of Management.. http://www.cphps.org/papers/phillips_econofethics_sep00.pdf.

[14] World Bank (2003). World Development Report 2004. Making services work for poor people.

